# Editorial: Biomaterials in Asia

**DOI:** 10.3389/fbioe.2023.1136139

**Published:** 2023-01-17

**Authors:** Yuce Li, Yin Fang, Mingqiang Li, Jianxun Ding

**Affiliations:** ^1^ College of Life Science and Health, Wuhan University of Science and Technology (WUST), Wuhan, China; ^2^ School of Chemical Engineering, College of Engineering, Sungkyunkwan University, Suwon, South Korea; ^3^ School of Chemistry, Nanyang Technological University, Singapore, Singapore; ^4^ Laboratory of Biomaterials and Translational Medicine, Center for Nanomedicine, The Third Affiliated Hospital, Sun Yat-sen University, Guangzhou, China; ^5^ Key Laboratory of Polymer Ecomaterials, Changchun Institute of Applied Chemistry, Chinese Academy of Sciences, Changchun, China

**Keywords:** biomaterial, drug delivery, tissue engineering, regenerative medicine, disease diagnostics

## Biomaterials in Asia

Biomaterials have shown significant potential in various biomedical fields, including disease prevention, theranostics, and tissue regeneration by controlled delivery of therapeutics, contrast agents, sensing agents, growth factors, and so forth. In recent years, Asian scientists have contributed more to biomaterials with the increasing support of various funds. Through this Research Topic, we expected to collect several state-of-the-art advances in biomaterial science and engineering research within Asia and disseminate them to the global audience. This Research Topic comprised 35 articles (including 7 reviews, 27 original research articles, and 1 case report) contributed by 263 authors, and it has attracted 54,023 views, 11,892 downloads, and 27 citations by researchers worldwide up to 15 January 2023. Undoubtedly, this Research Topic has won great success in displaying the significant contributions of Asian scientists to the next-generation precision medicine and further clinical translation.

The reviews from this Research Topic have covered the latest progress in several different types of biomaterials for diverse biomedical applications, including intraocular lens materials and their surface modification for cataract surgery (Luo et al.), drug delivery systems for the treatment of autoimmune diseases (Li et al.) and intervertebral disc degeneration (Liu and Fu), and engineering exosomes for bone defect repair (Ma et al.). Also, anti-biofouling polymer materials with special surface wettability and their biomedical applications are summarized (He et al.). Particularly, this Research Topic included two review articles about the recent development of organ-on-a-chips, regarding the application of medical imaging methods and artificial intelligence (Gao et al.) and physiologically based pharmacokinetic (PBPK) modeling (Yang et al.). These well-organized reviews would provide the readers with an overview and constructive perspectives of these specific topics of biomaterials.

The original research articles on this Research Topic have contributed to several critical aspects of biomaterials, including the preparation, characterizations, biological functions/mechanisms, and practical applications in clinic.

Smart biomaterials have shown distinct and adjustable *in vivo* behaviors for the delivery of therapeutic agents, such as small-molecule drugs, genes, and proteins, which is promising to improve the limitations of such therapeutics in clinic, including the low solubility/stability, short circulation, unsatisfactory selectivity on target tissues, and so forth. For example, cancer cell membrane-coated biomimetic mesoporous organosilica nanoparticles improved the anticancer performance and decreased the *in vivo* system toxicity of cisplatin because it prolonged the circulation time and increased tumor accumulation (Chen et al.). Jiang et al. demonstrated that micelles containing 15-crown-5 selectively released the loaded curcumin in response to the intracellular potassium ion (K^+^) by forming the 2:1 ‘‘sandwich’’ host-guest complexes between 15-crown-5 and K^+^. Similarly, an amphiphilic micelle comprised of a Y-shaped polypeptide has demonstrated excellent drug loading and release behaviors (Hua et al.). Interestingly, a carrier-free nanomedicine composed of ginsenosides Rg3 and Rb1 has shown better antitumor and antimetastatic effects against triple-negative breast cancer than the direct combination of free drugs (Zuo et al.). In addition, a cyclic Arg-Gly-Asp (cRGD)-decorated liposome loading with urokinase has shown enhanced thrombolytic effects by active targeting delivery of urokinase to the thrombi (Li et al.). Sun et al. developed electroactive shape memory polymers based on polyurethane and carbon nanotube, which would be employed as four-dimensional (4D)-printing materials for potential biomedical applications.

Some biomaterials have shown capacities to manipulate the behaviors of cells. For example, graphene was proven to promote the differentiation of Lgr5+ progenitors into inner ear hair cells (Ding et al.). Besides, copper-lithium-doped nanohydroxyapatite promoted the migration and homing of mesenchymal stem cells (MSCs) by upregulating the hypoxia-inducible factor 1α/stromal cell-derived factor-1 (HIF-1α/SDF-1) pathway (Li et al.). Interestingly, increasing the viscoelastic properties of cellulose nanocrystal/collagen hydrogels promoted the proliferation, alteration of shape, and matrix deposition of chondrocytes and reduced the interleukin-1β (IL-1β) secretion (Liu et al.). Moreover, Xia et al. developed magnetic nano chains, which guided the oriented growth of neural stem cell-derived neurons. Zhang et al. demonstrated that a conductive hybrid hydrogel promoted the development of neural stem cells into neurons. Wang et al. indicated that epigallocatechin-3-gallate selenium nanoparticle scavenged reactive oxygen species (ROS) effectively and thus provided superior neuroprotective effect. Qin et al. demonstrated intimate interactions among innervation, angiogenesis, and inflammation in the condylar cartilage of temporomandibular joint osteoarthritis, which provided promising treatment targets. In addition to developing advanced biomaterials, the surface modification of materials is also a method to obtain biomaterials with specific functions. For example, coating 316L stainless steel with glycogen synthase kinase-3β inhibitor (GSKi) improved the adhesion and proliferation of human coronary artery endothelial cells (Zhang et al.). Such properties of these biomaterials made them promise to treat various diseases, such as sensory hearing loss, osteonecrosis, osteoarthritis, neurodegenerative disorders, cerebral ischemia-reperfusion injury, and cardiovascular diseases.

In the past several decades, tissue engineering and regenerative medicine have emerged as essential branches of biomaterials. Several impressive research articles have provided constructive insights into the current Research Topic. Fu et al. performed a systemic bibliometric and visualized analysis regarding the photosensitive hydrogels for tissue engineering. Qin et al. established a “click” reaction-based method for surface modification of polycaprolactone scaffold, which could be utilized in the long-term controlled release of multiplex signal proteins for tissue engineering. Zhang et al. developed a polydopamine-poly(lactic-*co*-glycolic acid) (PDA-PLGA) scaffold carrying RINm5f islet cells, which supported the growth of islet cells and showed no influence on insulin secretion. When the type I diabetic rats were transplanted with the scaffold in the skeletal muscles, their blood glucose was maintained at a low level for approximately 3 weeks.

As emerging multipotential natural biomaterials, exosomes and secretomes have exhibited bright prospects in regenerative medicine. Particularly, scaffolds containing engineered exosomes derived from adipose-derived mesenchymal stem cells (A-MSCs) under hypocapnia (Wang et al.) and hypoxia-pretreated MSCs (Liu et al.) or secretomes derived from basic fibroblast growth factor (bFGF)-pretreated human umbilical cord blood-derived mesenchymal stem cells (hUCB-MSCs) (Liu et al.) have significantly facilitated the neural regeneration, which demonstrated great promise in the treatment of traumatic brain injury and the functional recovery after surgical treatment.

Biomaterials are also developed as sensitive detectors or contrast agents for disease diagnostics and identification of microbes/biomolecules. For example, Lin et al. reported polydopamine-coated silicon quantum dots as fluorescent probes of bacteria and their biofilms. Selective labeling of Gram-positive and Gram-negative bacteria was achieved by altering the surface functionalization groups. Besides, Liu et al. developed a visual and temperature-sensitive probe to detect microRNA, which could be biomarkers of diverse diseases like cancer. This biosensor enables point-of-care detection of biomolecules with low cost, ease of operation, and high sensitivity.

Clinical translations and applications are the terminal goals of development of biomaterials. The unique or modified biomaterials and biotechnologies should benefit more patients only when they pass the reliability assessment and model validation. Sun et al. provided an updated design of percutaneously osseointegrated prostheses for amputees, in which new bone formation at the bone-implant-skin opening area and distal bone canal was observed. For dental materials, Liang et al. demonstrated that the hot etching of zirconia with hydrofluoric acid (HF) induced a more uniform and dense porous morphology, greater roughness, and provided the highest shear bond strength. In addition, a multicenter retrospective study has demonstrated the high efficiency of ultrasound-guided percutaneous thermal ablation in the clinical treatment of hepatic focal nodular hyperplasia (Yu et al.).

Notably, the severe side effects of some medicines have limited their broad applications, although they are approved for clinical use. This Research Topic also reports strategies to reduce the side effects of clinically used therapeutics. Lan et al. developed acitretin-conjugated dextran nanoparticle (ACT-Dex NP), which achieved a similar therapeutic effect on psoriasis-like skin disease at a significantly lower dosage compared to neat acitretin. This low dosage is promising to avoid acitretin’s well-known teratogenicity on fetuses. Moreover, Zhang et al. demonstrated that the neomycin-induced ototoxicity would be prevented by combination with tetrandrine (a bioactive bisbenzylisoquinoline alkaloid derived from *Stephania tetrandra*) by promoting the steroid biosynthesis.

This Research Topic also included a rare case report in clinic (Wang et al.). The authors reported a patient with an uncommon pseudoaneurysm, involving the left common iliac artery because of brucellosis. A favorable therapeutic effect and well prognosis were obtained with a combination treatment of long-term multi-course antibacterial therapy with combination antibiotics. This report would provide a paradigm for the clinical treatment of such diseases.

In summary, this Research Topic encompasses a proportion of critical contributions made by Asian researchers in the biomaterial field in recent years. This regionalized Research Topic showed the rapid development of biomaterials in Asia, and it is foreseeable that Asian scientists will play a more significant role in this field in the future.

